# Assessing water distribution and agricultural expansion in the Cele Oasis, China

**DOI:** 10.1007/s10661-020-8233-2

**Published:** 2020-04-15

**Authors:** Brian Waldron, Dongwei Gui, Yi Liu, Lydia Feng, Heng Dai

**Affiliations:** 1 University of Memphis, Memphis, TN USA; 2 Xinjiang Institute of Ecology and Geography, Urumqi, Xinjiang China; 3 Jinan University, Guangzhou, Guangdong China

**Keywords:** Oasis, Food security, Agriculture, Water availability, Scarcity

## Abstract

Oases support 90% of the province’s inhabitants and produce more than 95% of the social wealth in Xinjiang Province of China. Oases’ dependency on water availability from mountainous regions plays a critical factor in the sustainability of agricultural practices and oasis expansion. In this study, we have chosen the Cele Oasis located in the south rim of the Taklimakan Desert, typical of oases in the region, as a case study to examine water availability. With over 97% of Cele’s economy tied to agriculture, unfettered expansion of the oasis into the desert has raised concern on water availability. A spatial and temporal analysis of water availability is performed using newly available data to determine whether agricultural production within the Cele Oasis has overexploited available water resources or if feasible expansion of agricultural production is feasible beyond its current boundary. Transferability of the methodology for assessing water availability spatially and temporally will be beneficial to other oases in the arid region that face similar concerns.

## Introduction

Water is essential to the formation and sustainment of oases in arid environments (Guo et al. 2016). Oases, naturally thriving yet fragile systems, have increasingly become locations of human habitation and modification (Ling et al. 2013; Liu et al. 2018). With essentially no other obtainable natural resources besides semi-fertile soil and water, agriculture is the predominant transformation to many oases (Song and Zhang 2015). Agriculture drives the local economy, which in turn heightens the desire to increase production, creating a virtually endless growth cycle; however, water availability sets an implicit limit to unfettered expansion (Bai et al. 2014; Sun et al. 2019). Determination of maximum growth can be problematic owing to a variety of external (e.g., climate change, politics, economy) and internal factors (e.g., land use, crop type, environment). However, it is possible to obtain a relative measure of oasis sustainment through a mass balance allocation approach (Ling et al. 2013; Xue et al. 2017).

This investigation focuses on the Xinjiang Province in northwest China where oases have had a long history. Earlier than 3000 years ago, incipient farming existed along lower river reaches in the Tarim River Basin where hand-dug canals easily transported water due to the near flat topography (Hong et al. 2003; Wang et al. 2013). Over 2000 years ago, Silk Road trade routes connected through oases in the Taklimakan Desert (Bi et al. 2014). Relied upon by traders and traveling merchants for water, these oases served a vital role in supporting shared economic prosperity between Asian and Western European civilizations. In this period of trade and discovery, oases were not just important watering stops for travelers, some also were population centers of a mix of ethnic cultures near the region (Lawler 2009; Mächtle et al. 2019). Even today, the oases serve a vital role in China’s economy and the indigenous population, yet they are not necessarily the same oases as before. In arid regions, small changes in climate can have dramatic and sometimes devastating effects on oasis structure and stability, especially when mixed with anthropogenic factors (Xu et al. 2004, 2006; Chen et al. 2007; Hao and Xu 2008; Lyu et al. 2016). The devolving of oases from their natural state into meccas of agricultural production has strained the finite water resources, in some cases to an extreme that the propagation of downstream effects has resulted in detrimental loss to desert oasis habitats (Xu et al. 2006; Chen et al. 2007; Zhou et al. 2016). The appeal to increase the economic gain by expansion of oasis agricultural production diminishes oasis sustainability, thereby necessitating the determination of available water resources and their allocation (Ling et al. 2013; Gao et al. 2018).

The Cele Oasis located along the southern rim of the Taklimakan Desert is typical of oases in the region. Since some of the earliest records in 1956, the Cele Oasis has expanded its agricultural footprint at a moderate pace (by a factor < 2.7) as compared with other oases in the area (Tian and Song 1997; Bruelheide et al. 2003; Qi et al. 2005; Xue et al. 2016; Liu et al. 2018). One might conclude that this transformation by most oases (termed “oasification”) has resulted in desertification; however, the Taklimakan Desert has witnessed a reversal due to rehabilitation efforts (Wang et al. 2008; Zhang et al. 2017). As a typical oasis in the region, the Cele Oasis has been expanding with a mindset of reducing anthropogenic effects under the assistance of Cele National Station of Observation & Research for Desert-Grassland Ecosystem (CLD) operated under the Chinese Ecosystem Research Network (CERN). The authors believe that the Cele Oasis offers an excellent opportunity to assess expansion possibilities. Thus, this research attempts to ascertain the plausible agricultural expansion in the Cele Oasis based on water contribution by snowmelt runoff from the Kunlun Mountains to the Cele River, diversion of surface waters to reservoirs, canal networks and desert outflows, groundwater withdrawals, and crop demand.

## Study area

Located in northwest China and known for its irrigation agriculture, the Xinjiang Province constitutes one-sixth of China’s mainland area. Within the Xinjiang Province is the Taklimakan Desert which is the world’s second largest shifting sand desert. The Taklimakan Desert is 377,000 km^2^ and is flanked by the Tienshan, Pamir, and Kunlun Mountains to its north, west, and south respectively, and abuts the Gobi Desert to the east. Residing inside the Taklimakan Desert is the Tarim River Basin (TRB) covering the entire southern part of Xinjiang.

Cele Oasis population has nearly doubled over the past 45 years from 87,000 in 1973 to 150,000 in 2018 (Bruelheide et al. 2003). In Cele’s early agricultural development, the Cele River was dammed in 1960 to redirect water northward for irrigation purposes. Consequently, this reorientation of surface water destroyed large stands of natural desert vegetation (e.g., *Populus euphratica*) triggering desertification which by 1980 had resulted in sand dunes near the town center and arable losses of 25% (Bruelheide et al. 2003. The presence of the CLD in Cele and its recommendations for desert rehabilitation reversed consequences of the Cele River dam. In 1987, a canal network composed of 33 km of a canal and 153 sluice gates diverted Cele River water throughout the oasis. The original earthen canal network was completely abandoned, with the new restructure following a fishbone pattern (Graefe et al. 2004). The new concrete canal system has allowed Cele to thrive as an agricultural community with over 90% of its footprint as arable.

**Fig. 1:**
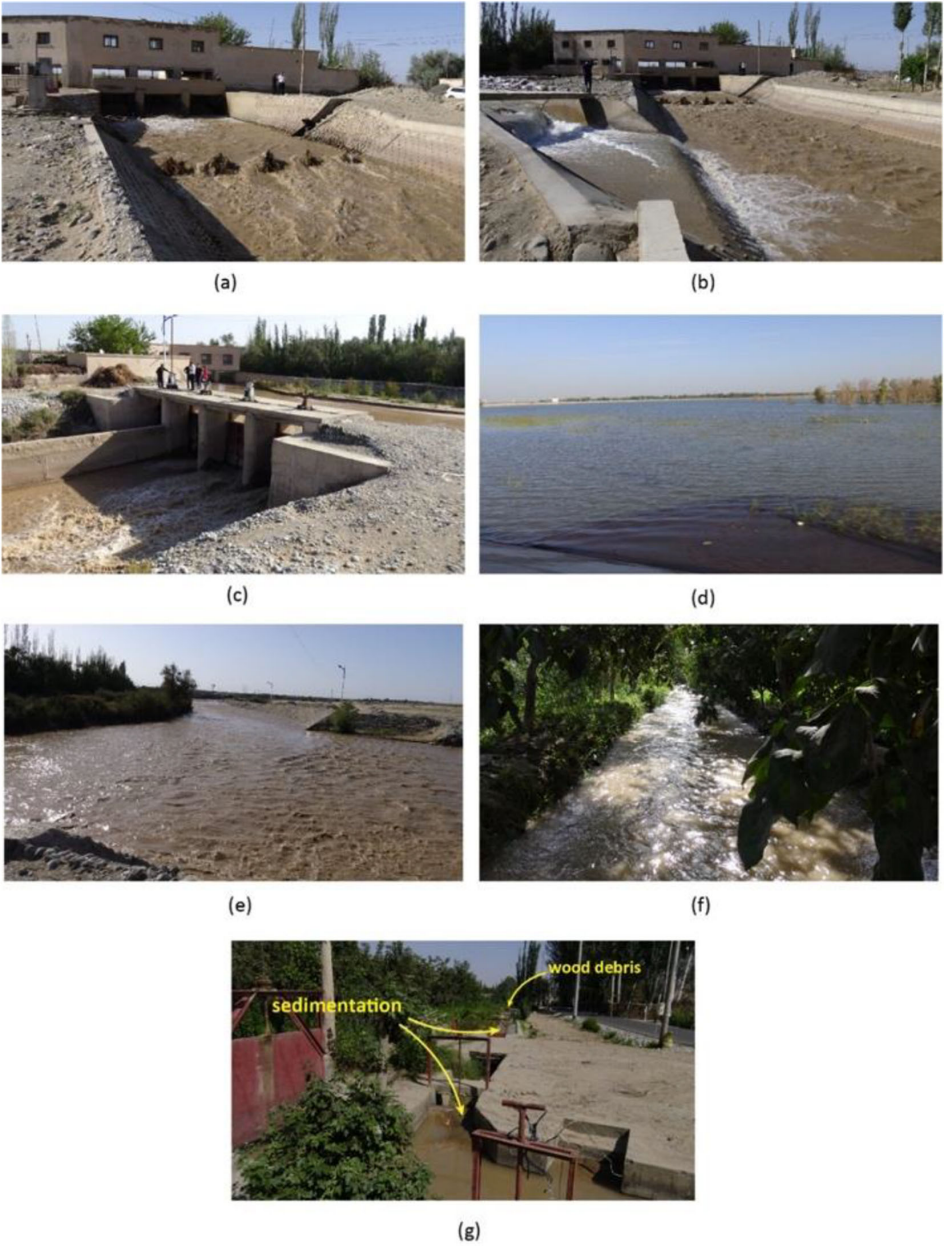
Cele gate and canal system (2017). (a) Outflow at gate 1 into main Cele canal channel. (b) Inflow from Cele reservoir into main canal channel. (c) Cele River in background with outflow through gate 1 side structure toward Cele reservoir. (d) Cele reservoir. (e) Overflow of Cele River across submerged broad-crested weir into the desert. (f) Concrete lateral carrying diverted water from gate 4 with trees providing shade. (g) Sedimentation clogging a gate to a north-south concrete lateral with sedimentation (not water) visible in the channel into the background and wooden debris filling the channel

The Cele Oasis’ primary water source is the Cele River. Though small farms dot the river’s banks and some diversion into alternate streams occur, a large majority of the river’s flow continues toward Cele, past a manually operated gaging station (8 km from Cele) before reaching the main diversion gate at the southern tip of the oasis. This is a government-managed gauge, and past surface water data from this gate were analyzed in this paper. The main gate has three diversion structures: (1) three entry gates into the oasis’ concrete canal network; (2) three gates exiting flow into a natural channel that supplies the Cele reservoir (see Fig. 1(c), (d)); and (3) a broad-crested weir that allows flood water to spill into a natural channel exiting to the west and north of Cele into the desert.

The Cele canal network is a series of concrete trapezoidal channels where laterals bifurcate off a main channel that runs through the middle of Cele in the north into the desert. These east-west laterals extend outward toward the fringes of the oasis conveying water through gates into a denser network of north-south channels. Along each north-south channel, farmers draw water into hand-dug earthen channels to carry water to their crops. Large gate structures control the release of water into the laterals stemming off the main channel. There are eleven gates, with the largest being gate 1 at the entrance to Cele where the gate structure is approximately 11 m wide (see Fig. 1(a)). Progressively getting smaller moving north, gate 11 and its laterals have a top width of 1 m. The canal network was designed to distribute captured flow from the Cele River throughout the oasis more effectively. However, the Cele River is prone to large amounts of suspended sediment where in a series of gates and channels, sedimentation is clogging portions of the network. Some farmers will keep channels clear of sediment and debris, but in other instances, channels remain blocked and lateral gate structures are permanently damaged (Fig. 1(g)). As the delivery of surface water into the oasis becomes more restricted, farmers rely on groundwater to supplement loss in surface water flow.

## Data and analysis

A mass balance of surface water to the Cele Oasis depends on accurate measurements of discharge at the Cele Oasis main diversion gate, which have recently become available. This section will explain the data collection and analysis methods that were employed in this paper.

### Surface water

**Fig. 2:**
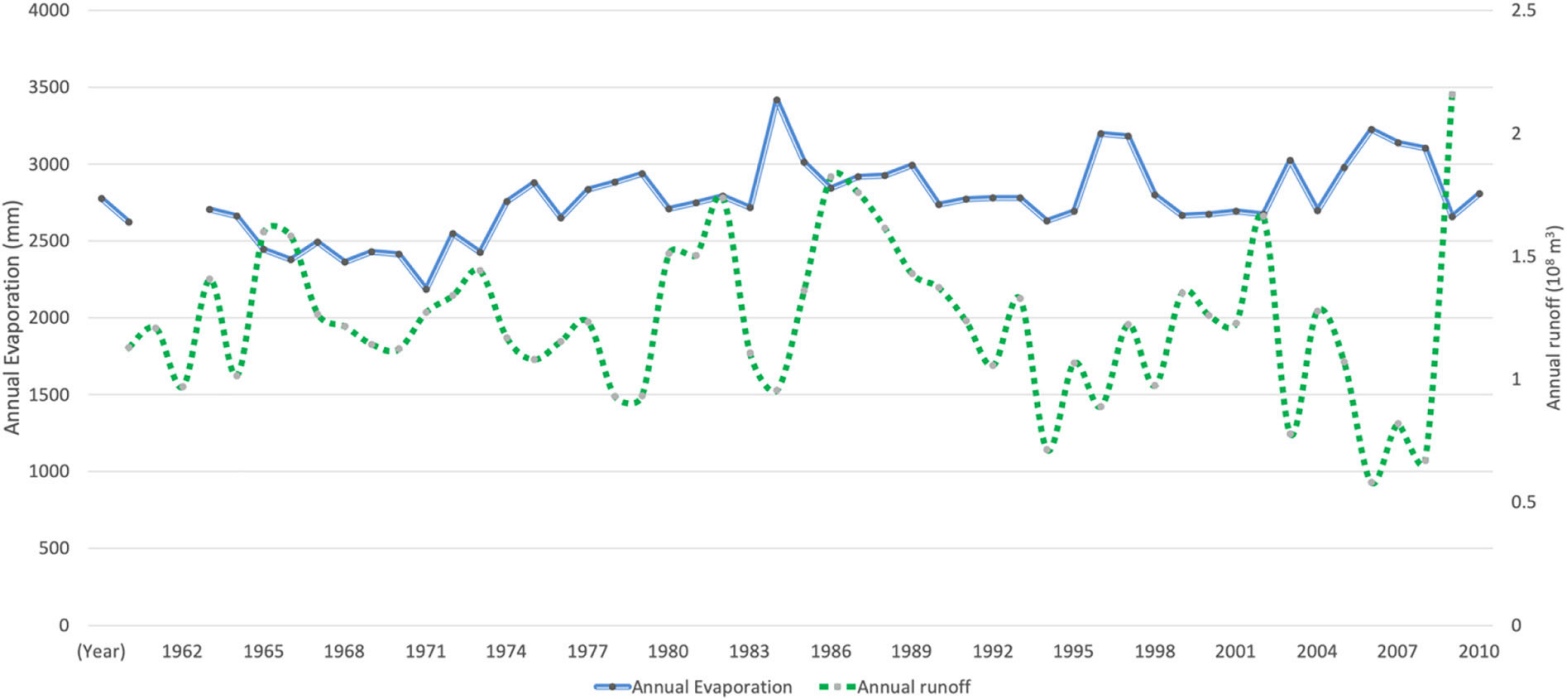
Annual evaporation and runoff in Cele Oasis from 1960 to 2010

Located at 37^o^ 0.95′ N by 80^o^ 43.75′ E at an altitude of 1319 m, the Cele Oasis heavily relies on runoff to the Cele River from the Kunlun Mountains 25 km to its south, originating as snowmelt and orographic rainfall (Hao and Xu 2008). As shown in Fig. 2, runoff varies from enormous flows during June–August to minimal outflow during December–February (Liu et al. 2019). Governing hydrologic processes in the mountains and desert can be described as precipitation-runoff and runoff-evaporation, respectively (Han et al. 2009). For the majority of the years from 1960 to 2010 in the Cele Oasis, the annual precipitation is < 50 mm (Cheng 1997; Li et al. 2011; Liu et al. 2018; Zhou et al. 2018) and pan evaporation is in the range of 2100–3400 mm (Fig. 2). Temperatures range between 41.9 and − 23.9 °C with an annual average of 11.9 °C (Chang et al. 2015).

**Fig. 3:**
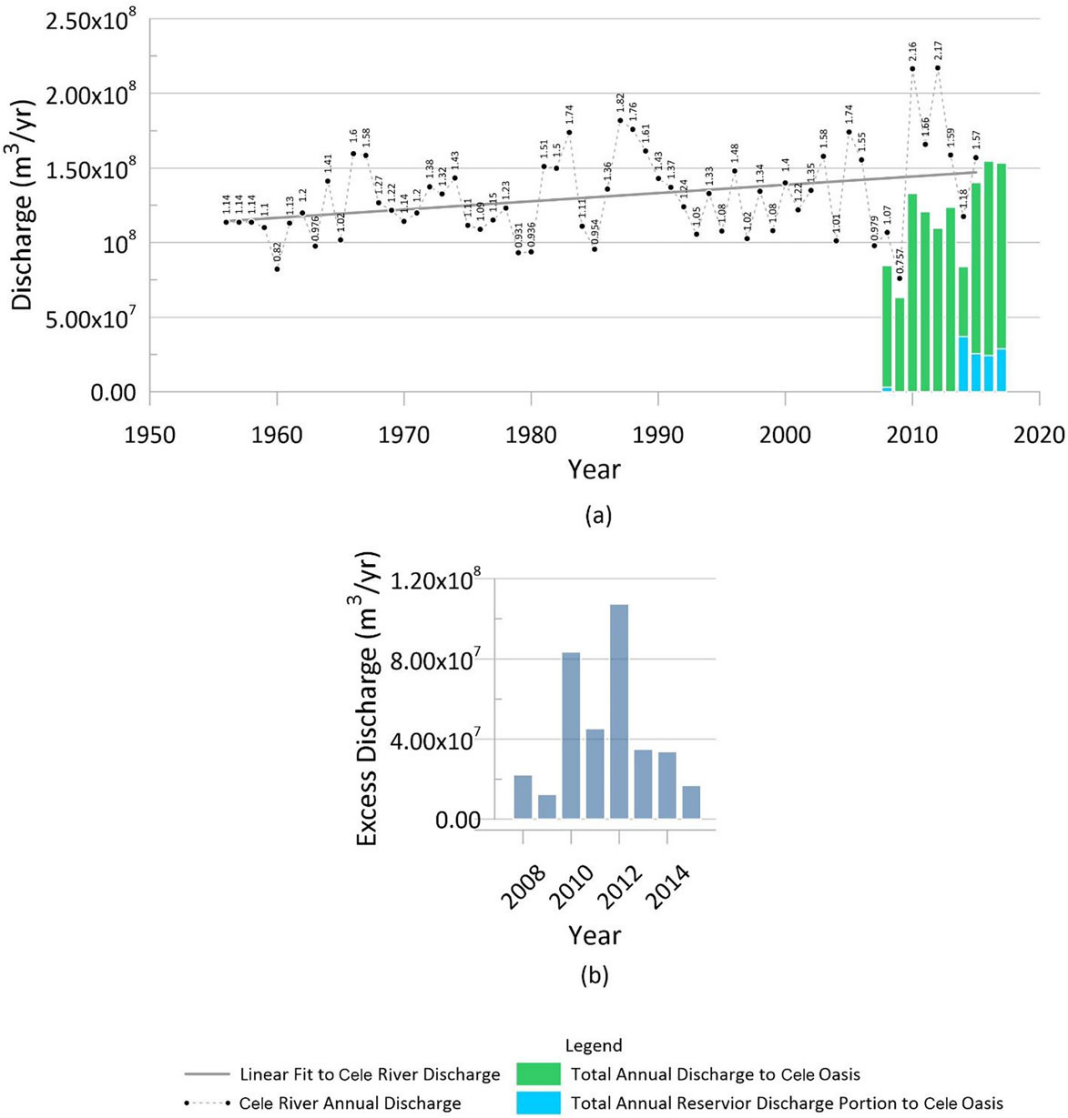
(a) Annual Cele River discharge (1956–2015) and diversion of flow into the Cele canal system (2008–2017). (b) Excess discharge by Cele River (2008–2015)

Cele River discharge is recorded daily at a gaging station approximately 8 km upstream of Cele. Though measured daily, only annual discharge records have been released. It is shown in Fig. 3 the Cele River discharge (m^3^/year) from 1956 to 2015. Variation in discharge is directly related to snowmelt runoff. Periods of drought (4+ years of consistent decline) are observed during the periods 1966–1969, 1987–1993, and 2006–2009, in which the Cele River had a record low discharge of 75.75 × 10^6^ m^3^/year in 2009. Climatic records indicate the Tarim Basin is getting wetter (Li 1991; Hong et al. 2003; Hao and Xu 2008) which corroborates the slight upward trend in Cele River discharge (Fig. 3). Daily discharge records also exist for gate 1 at Cele, beginning in 2008; however, completeness of recordings varies among years. In 2018, daily discharge data for flows at gate 1 (Fig. 1(a)) were obtained for the period of January 2008 to May 2018. Daily discharges through gate 1 into the Cele main arterial canal were consistent during the reporting period. Measure of discharge to the Cele reservoir began in 2008, but remained uncalculated until 2014 when they were reinitiated (Fig. 1(c, d)). Sporadic measures of flood water diverted into the desert exist (Fig. 1(e)), but there is not enough information on its calculation nor reliance in the consistency of measure to allow for a valid assessment of that flow.

As shown in Fig. 3(a), diversion of flow into the Cele canal network has not exceeded river discharge, but it is impacted by periodic declines in snowmelt runoff, for example, as in 2009 and 2014. The portions of flow to the canal network from the Cele reservoir are indicated. Using records from 2014 to 2017, reservoir contributions accounted for 21.8% of total flow on average. Between 2008 and 2015, there was an average excess of 44.5 × 10^6^ m^3^/year; however, flows in 2010 (216.3 × 10^6^ m^3^/year) and 2012 (217.0 × 10^6^ m^3^/year) produced elevated excesses of 83.4 × 10^6^ m^3^/year and 107.4 × 10^6^ m^3^/year, respectively, causing the average to be deceptively beneficial when considering the potential for expanded growth as the average is greater than calculated excesses for 5 of the 6 remaining years (2008, 2009, 2013–2015). 2011 excess was barely above the average at 45.2 × 10^6^ m^3^/year. When considering use of excess discharge for oasis expansion, policymakers need to consider time of concentration which is problematic to derive with only annual Cele River discharge data and, if held in storage, must consider evaporation and infiltration loss. However, daily discharge readings at gate 1 may serve as an excellent proxy to Cele River annual discharge readings. In fact, Cele River flow across the 8 km distance would lessen owing to the two stressors mentioned above, and this gives better credence to using gate 1 readings with cautions of some unknown or absent diversions (e.g., flood waters to the desert, reservoir release to the desert, and canal outflow past gate 11 into the desert).

**Fig. 4:**
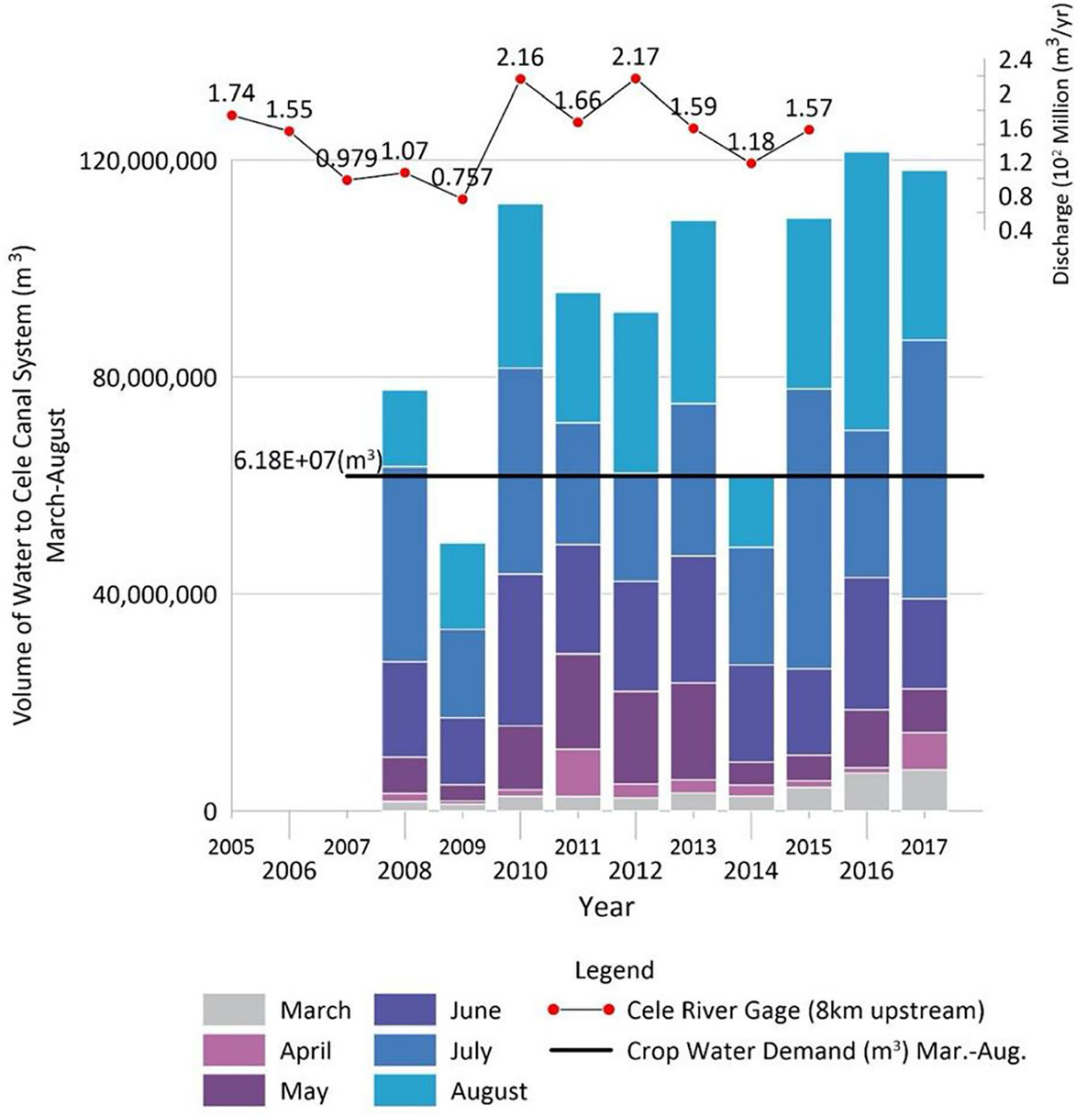
Contribution of surface water to Cele canal system during the growing season (March–August)

Most of the water consumption in Cele Oasis occurs during the growing season which is March–August. As more than 90% of Cele is devoted to agriculture, the use of surface water during this period can offer insight into water availability to crops and possible expansion. Figure 4 shows monthly water contribution (m^3^) to the Cele canal network from direct diversion through gate 1. Inflow from the Cele reservoir and additional contribution from the Cele reservoir is minimal (see Fig. 1) when compared with the other two sources (2014, 1.97 × 10^6^ m^3^; 2015, 0.795 × 10^6^ m^3^; 2016, 11.0 × 10^6^ m^3^; 2017, 6.63 × 10^6^ m^3^). Based on observations from gate 1, Cele River discharge begins to increase in March–May, but reaches its heightened flow during June–August. If expanding Cele’s agricultural production by transforming desert into cropland is considered, excess flow during this period will be of profound importance. The current total irrigation water demand is 6.18 × 10^6^ m^3^ (Fig. 3, discussed later). Except at the end of drought periods (2009) and flow decline (2014), surface water sufficiently meets agricultural demand. Chang et al. (2015) created evapotranspiration estimates for Cele and two other oases that account for infiltration loss through deep recharge occurring below the root zone. The crop type irrigation rates provided by the farmers implicitly account for evapotranspiration and infiltration loss. The Cele canal network is concrete so infiltration is minimal. Evaporation loss from the channels is unknown, but the mostly shaded main channel and its laterals from the gates provide some expected measure of reduced evaporation (Fig. 1(f)). Assuming minimal loss from the canal network, there is an annual average excess 32.9 × 10^6^ m^3^ (2008–2017)—this amount represents 53% of the present agricultural demand.

### Groundwater

The Cele Oasis has over 200 production wells that withdraw groundwater from an unconfined aquifer. Groundwater is used to supplement surface water during periods of low flow or in agricultural areas not connected to the canal network. In response to concerns over water shortages in northwest China, some wells have been retrofitted with meters to regulate flow (Chang et al. 2015); however, withdrawals are unregulated and unknown in Cele. The CLD has performed groundwater level surveys since 2008 and took observations from 25 selected wells distributed across Cele. Many surveys were conducted once or twice a year, with 2008 and 2010 as exceptions when they were performed monthly and bi-monthly, respectively. Often, surveys were conducted in June, the peak month for runoff from the Kunlun Mountains. No surveys were carried out in 2012 and 2013. In addition to these surveys, CLD has monitored groundwater levels in two research wells since 2005 and took observations three times a month on days 10, 20, and 30 (for February, measurements were taken on the last day) (Chinese Ecosystem Research Network 2005). Based on limited data sources, only a determination of drawdown patterns was made in this paper.

**Fig. 5:**
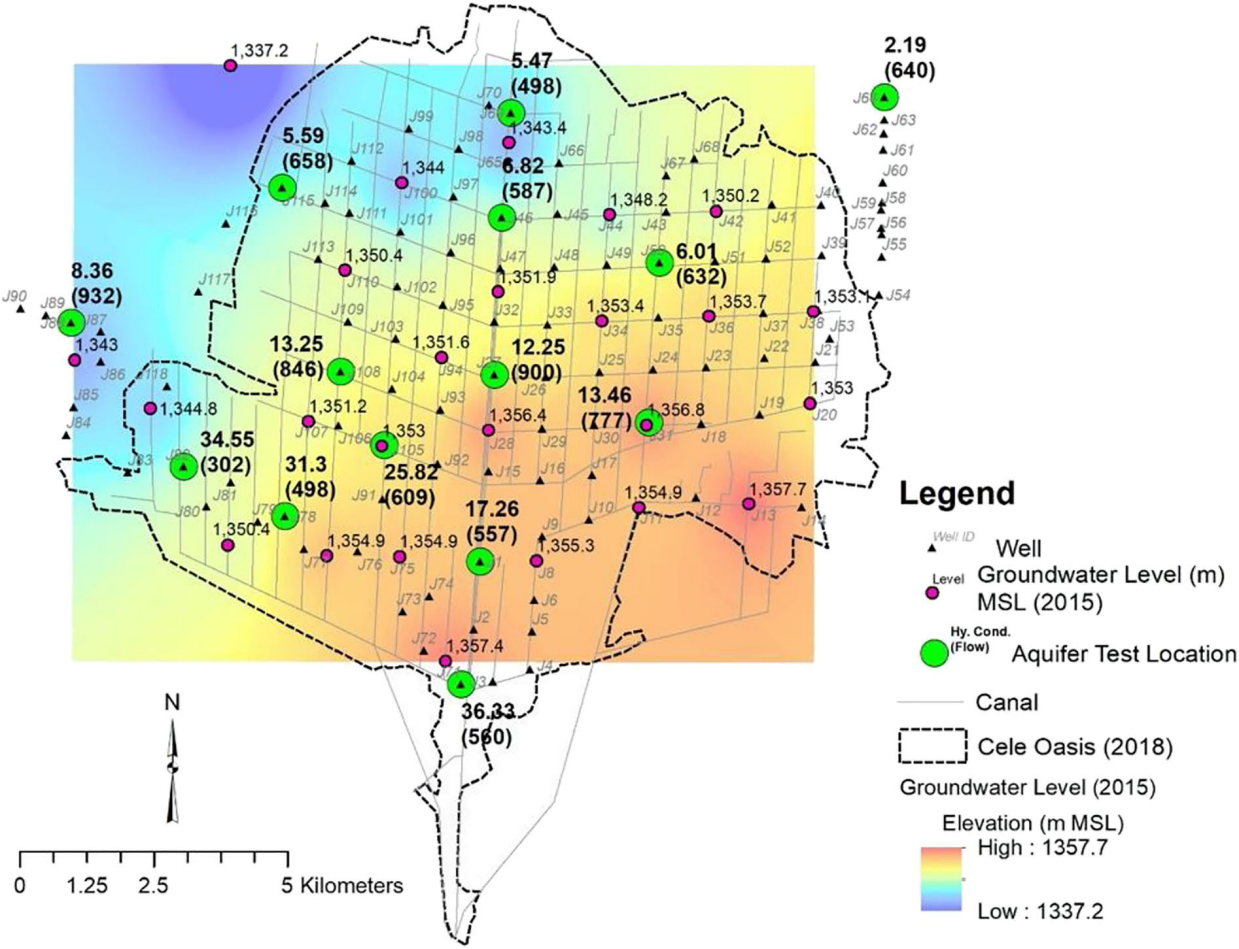
A sample number of groundwater wells in Cele including locations of aquifer tests for hydraulic conductivity (m/day) with withdrawal rate (m^3^/h) and 2015 groundwater level survey points with accompanying gradient surface. Interpolation of 2015 groundwater levels was performed using IDW

Cele’s reliance on groundwater comes from a vertical sequence of unconsolidated sediments composed of silty sand of 20–80 m thick followed by a deeper sequence of sand of 40–160 m thick. Groundwater wells are screened to depths of 90–120 m below ground surface (BGS). The groundwater is found between 63.84 and 2.38 m below land surface (BLS) with a mean of 18.2 m (2015), and a gradient southeast to northwest (Fig. 5). At the time of their investigation, Guo et al. (2008) stated that the groundwater depth in Cele was 16 m and 7 m on the headland. Liu and Wang (2013) measured hydraulic conductivity at 14 wells within the Cele area, substantiating the heterogeneity of the aquifer. In Fig. 5, conductivity in lower-central Cele ranges from 12.25 to 36.33 m/day, while outward to the north conductivity falls between 2.19 and 8.36 m/day. Withdrawal rates are similar among all the wells with an average of 295 m^3^/h. Not shown in Fig. 5 are four additional measures of conductivity that are between 1.22 and 2.7 m/day ($\overline{Q}$=127 m^3^/h), since their relevance to the aquifer underlying Cele is questionable. These additional measurements are 4–15 km northwest of Cele from deeper wells (200–225 m) that are constructed with 3–5 separate screened intervals and possibly penetrate a deep sequence of sand conglomerates. Geologic cross-sections (Liu and Wang 2013) indicate high unconformity between the silty sand and sand sequences; however, these sections fall outside of Cele which makes direct interpretation within Cele difficult. What can be inferred is that the two sequences of sediments are in hydraulic connection due to the absence of an intervening confining layer; therefore, analysis of drawdown and groundwater availability is based on the premise of this being a single hydrogeologic unit.

**Fig. 6:**
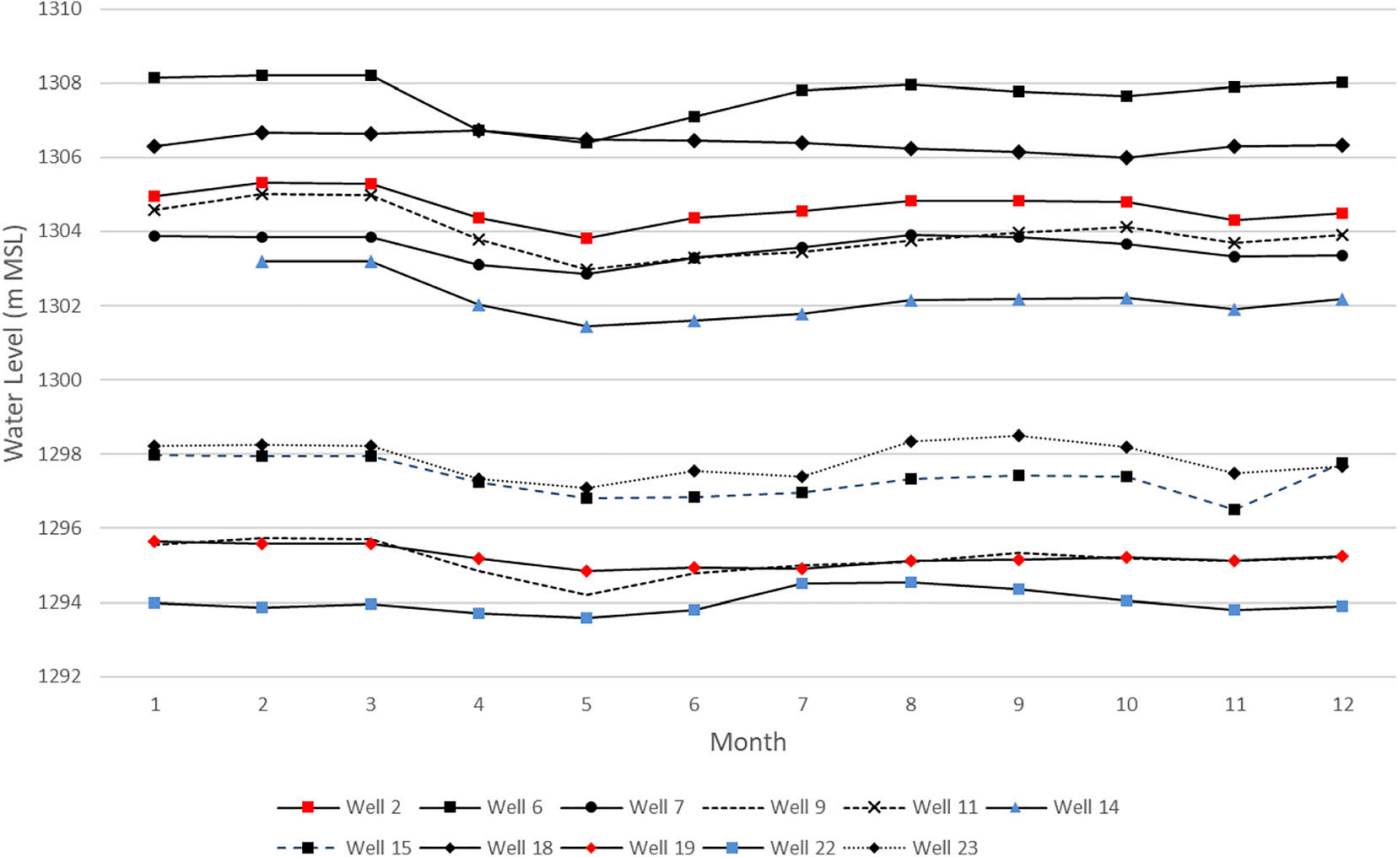
Monthly (Jan.-Dec.) groundwater levels from 11 observation wells during 2008

**Fig. 7:**
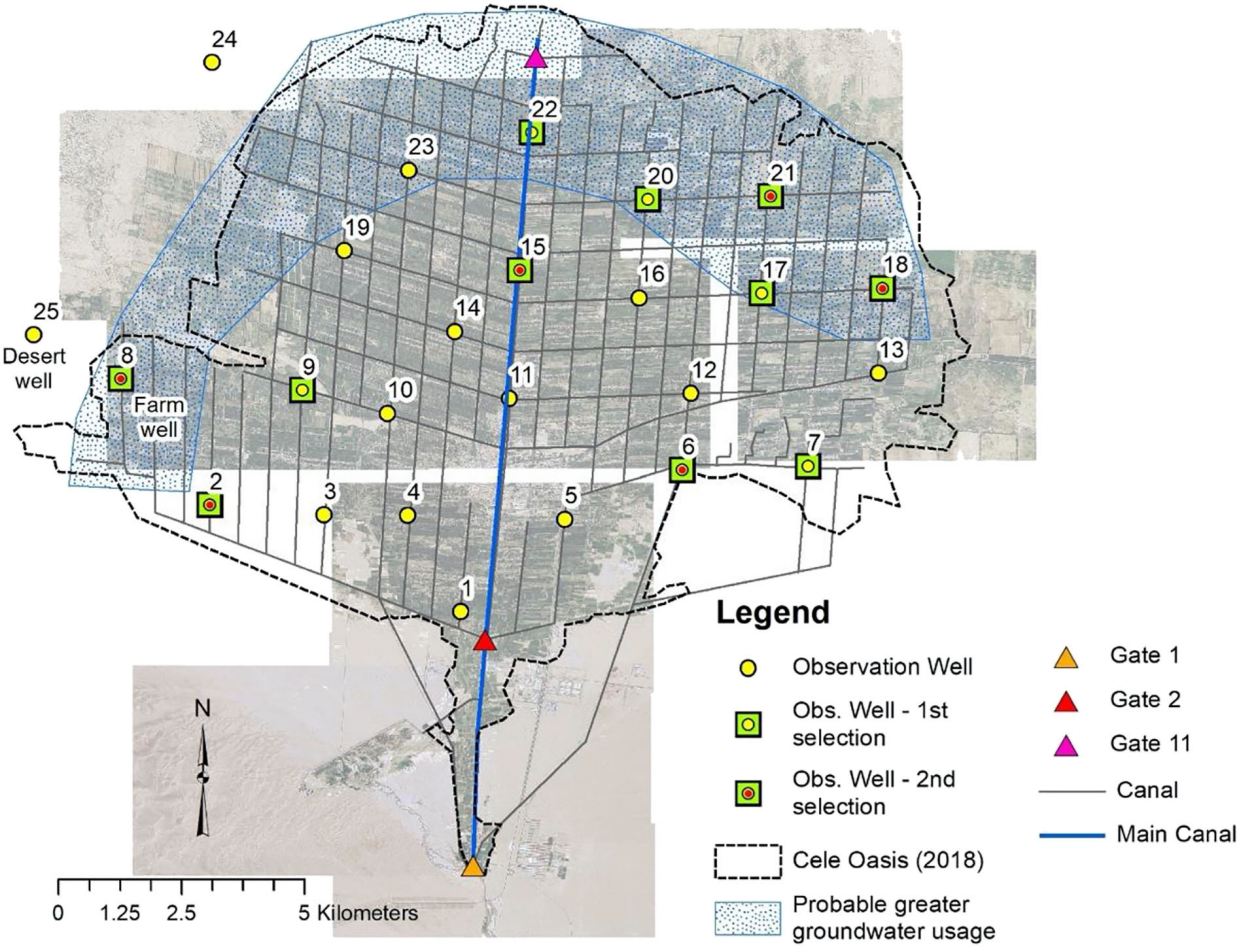
Observation well locations in Cele including locations of 1st and 2nd selection of observation wells for record of groundwater level decline during the period 2008–2015

Groundwater levels for as many as 25 wells were measured annually between 2008 and 2015, usually during the month of June (2008, 2009, 2010, 2011, and 2015), but also at other times during the year: 2008 (1–12); 2009 (10, 12); 2010 (2, 4, 6, 8, 10, 12); 2011 (2, 4, 6); and 2014 (8). An additional two wells located on the western outskirts of Cele at the Cele Desert Research Station had continuous readings from 2005 to 2014 three times a month. The finding from these two wells is discussed later in this section. Most recent water levels (2015) indicate a north-northwest gradient (Fig. 5) during June. Seasonal variations in groundwater levels were determined using 2008 and 2010 data when measurements from the first set of 25 wells were collected over the span of a year. During 2008, groundwater levels were measured each month for 13 of the 25 wells. Of these 13 wells, two (#3 and #8) had incomplete records. Investigation of 2008 groundwater levels from the remaining 11 wells reveals a muted seasonal effect during the months of March through August for the majority of Cele (see Fig. 6) except in well 18 where groundwater levels are observed rising during these months. However, this may be explained by well 18 being located on the eastern outskirts of Cele where agricultural development is not as pronounced (see Fig. 7). A second groundwater level depression is observed in November for 7 of the 11 observation wells (wells 2, 7, 11, 14, 15, 22, and 23) where levels are an average 31.4% less than those during March–August with the exception of well 15 which indicated a slightly larger drawdown (+ 0.3 m) during November.

During January–December of 2008, the overall drawdown of groundwater is considered minimal with a range of 0.76 to 2.02 m, with the smallest and largest drawdowns observed in wells 18 and 11, respectively. On Cele’s main canal at gate 1, the Cele River is diverted either into the canal system, overland flow to the Cele reservoir, or overland into the desert. Cele’s first main diversion gate is gate 2, with the main canal reducing in size at subsequent gates before terminating and discharging into the desert at the oasis’ northern end. As more surface water is available at gate 2 and along the main canal, it is plausible that groundwater is relied on more heavily as surface water availability from the canal network lessens (due to a smaller sized conveyance network) which can be approximated in the arched area in Fig. 7. An analysis is performed to determine if groundwater drawdown is related to distance from more prevalent zones of surface water. Using the measured drawdown ranges from 2008, drawdown is mapped by Euclidean distance from the main canal and gate 2 (Fig. 7).

**Fig. 8:**
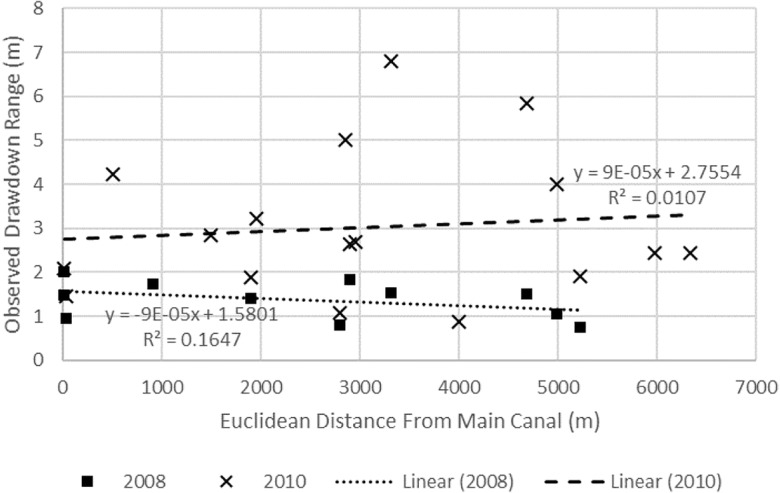
Relationship between 2008 and 2010 observation well locations and Euclidean distance from the Cele main canal

**Fig. 9:**
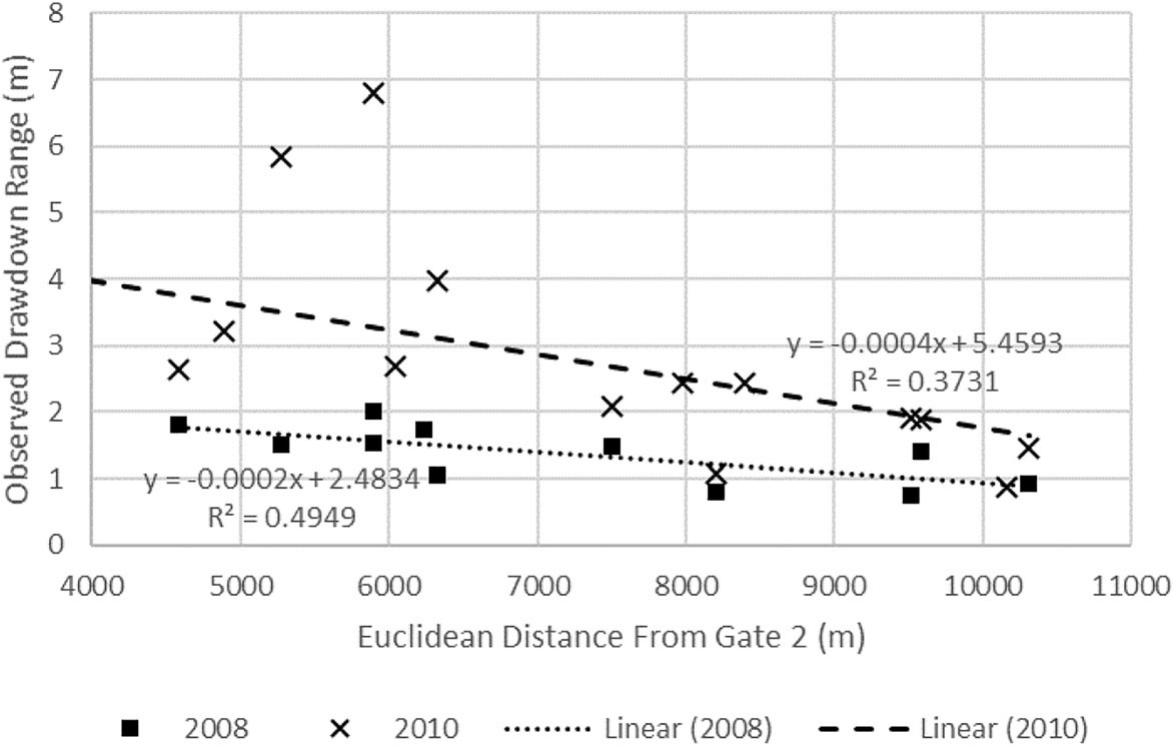
Relationship between 2008 and 2010 observation well locations and Euclidean distance from gate 2 where diversion of surface water laterally into the Cele Oasis begins

The measured distance from the main canal to the observation well reflects the distribution of water within the canal network where, as distance from the main canal increases, water become less available due to draw-off from laterals and progressive shrinking in the canal channel dimensions. Hence, as distance increases from gate 2 upward and outward into the Cele Oasis via the canal network, surface water conveyance quantities will diminish. As indicated in Fig. 8, the distribution of drawdown by distance is independent of distance from the main canal (*R*^2^ = 0.1647). It relates to distance from gate 2 more strongly (*R*^2^ = 0.4949), yet not enough to suggest groundwater drawdown is dependent on location (Fig. 9). A similar analysis was performed using 2010 observations, the only other period of record during 2008–2015 that has depth to water measurements (although only for 6 months starting in February and measured every other month). Drawdowns range between 0.87 m and 5.85 m, twice more than the average in 2008; however, as shown in Figs. 8 and 9, groundwater drawdown is independent of location. Therefore, there are no observable site-specific areas where groundwater withdrawals notably exceed or are dependent on surface water availability via the Cele canal network.

**Fig. 10:**
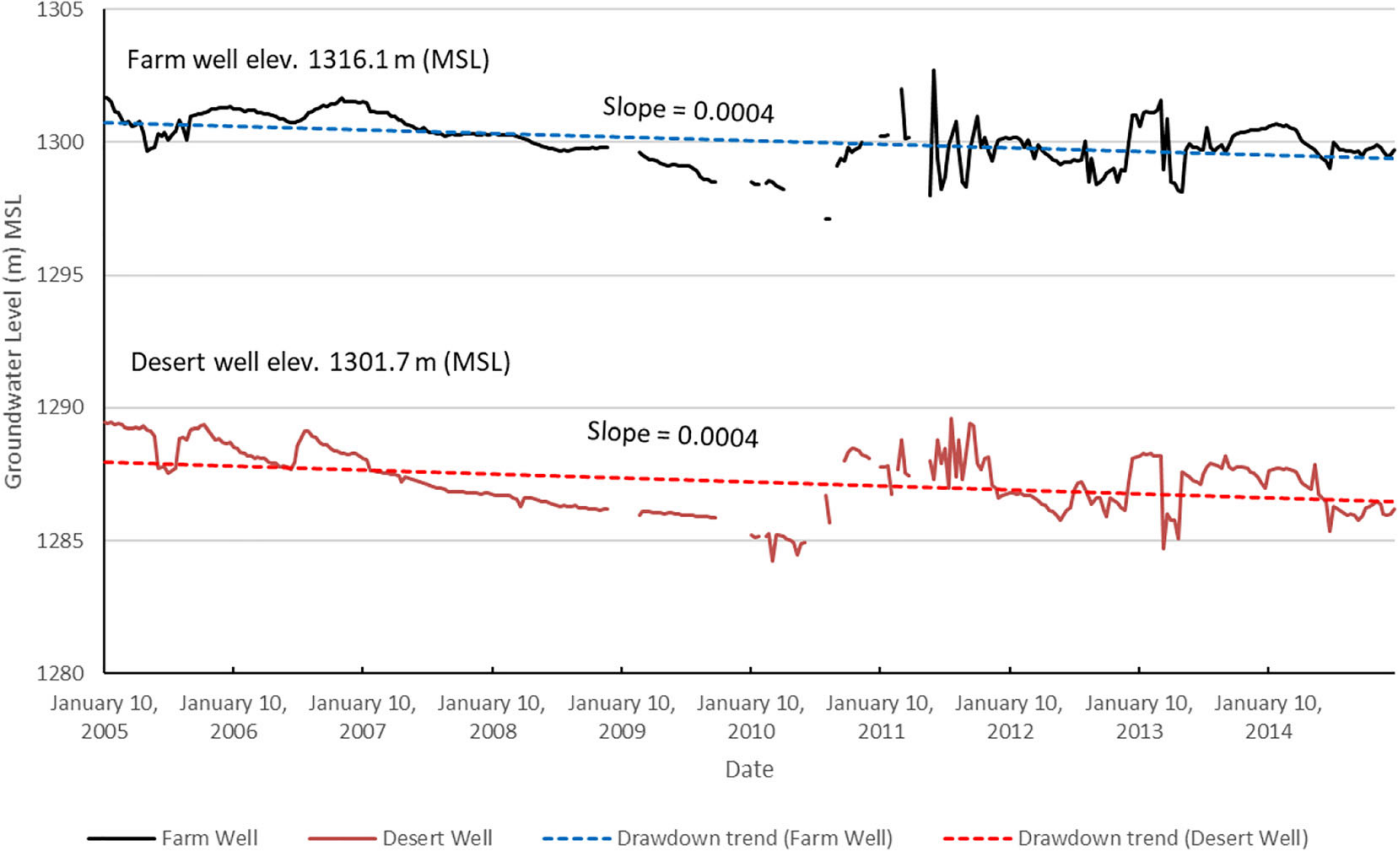
Recorded groundwater levels in two observation wells operated by the Cele Desert Research Station, Jan. 2005–Dec. 2014

As previously mentioned, the Cele Desert Research Station has monitored groundwater levels in two wells near its location along Cele’s western border. One well, called the farm well, is located near well 8 and the second well, or desert well, is located near well 25 (Fig. 7). Observations were made 3 times a month (10th day, 20th day, and 30th day) during 2005–2014. The groundwater responses in these two wells were investigated separately from the other 25 wells due to their difference in period of observation, their remote isolation (i.e., lack of broad distribution), and their detailed record (i.e., 360 recordings compared with a typical 12 with the other 25 (excluding year 2008)). Nevertheless, the readings from these two additional observation wells are of value. The decline of groundwater in this region (an area of significant water usage for agricultural irrigation) is not pronounced (see further discussion in the next section). As shown in Fig. 10, groundwater levels have been declining since 2005, yet the linear trends with a slope of 0.0004 indicate it is minimal. During the period of March–September 2011, there is noticeable oscillation on a 10-day periodicity as though a pump(s) is turning on and off. During 2012, there seems to be production from irrigation wells that drawdown the water level during the growing season of March–August, but a possible period of recovery in the off-season into winter, with a sudden drop in March 2013 when the agricultural production period resumes, however at a shorter time span than before into August. Unfortunately, due to the absence of production well records such as location, pumping rate, and time of withdrawals, a more complete assessment of these records is not possible.

**Fig. 11:**
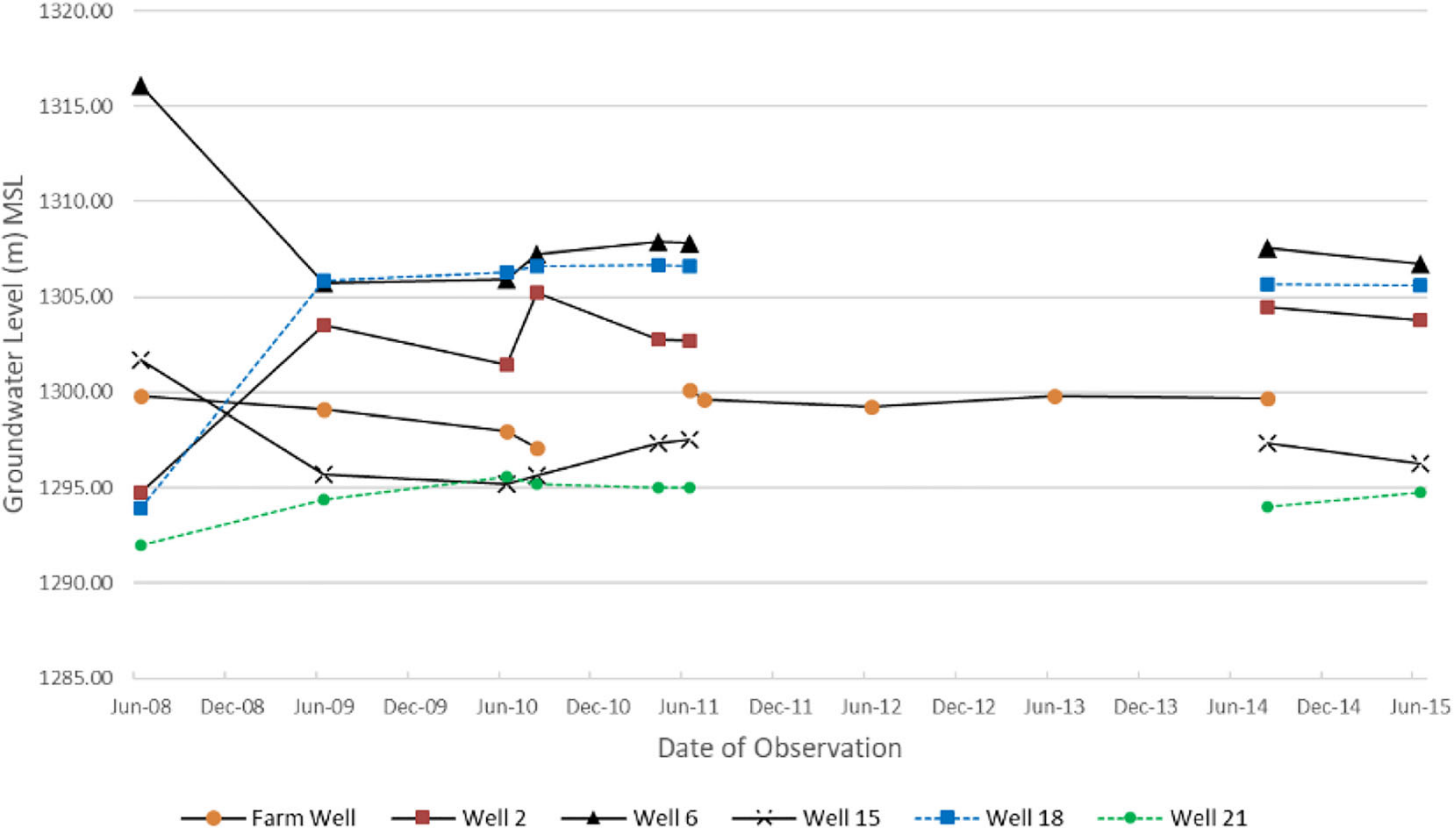
Observed groundwater levels in six selected observation wells during the period 2008–2015

To ascertain any trend in groundwater rise or decline since 2008 across the Cele Oasis, eleven observation wells were selected for their record of completion during the greatest drawdown period (March–August). Any significant declines in groundwater warrant concern for a trend toward depletion, yet also impacted are the desert ecosystems that depend heavily on such underground resources (Xue et al. 2015). Based on spatial proximity to the main canal and gate 2, six observation wells were chosen to represent historic water level trends (see Fig. 11). In particular, observed levels from the farm well were averaged over the same month as the other wells, but for missing periods of 2012 and 2013, farm well observations were averaged only for the month of June based on available data. With the exception of the farm well, missing groundwater level observations from 2012 and 2013 make a complete analysis more interpretive from 2011 to 2015 (Fig. 11). As shown in Fig. 11, moderate groundwater level declines of 10.33 m and 6.0 m are observed in wells 6 and 15, respectively, and a slight decline of 0.65 m in the farm well from 2008 to 2009. In contrast, during the same period, groundwater levels rose to 8.57 m and 11.92 m in wells 2 and 18, respectively, followed by minimal change through 2015. Well 21 had a moderate incline in the water table between 2008 and 2010 with minimal change through 2015. The notable declines and rises in water level between 2008 and 2009 as compared with 2009–2015 may be a function of Cele experiencing its fifth year of drought in 2009 (2009 Cele River runoff: 0.757 × 10^6^ m^3^). However, during an exceedingly large runoff in 2010 (Cele River runoff: 2.16 × 10^6^ m^3^) followed by continued exceedances in river discharge through 2015 (see Fig. 3), water levels in all six observation wells have a minimal fluctuation of 1.08–3.77 m (average = 2.21 m). This agrees with ShuYong et al. (2009) who observed groundwater level fluctuations in Cele of 0.23–2.25 m between April and September during 2004–2007. Impactful to this analysis but unknown are the number of production wells installed and abandoned during this 8-year period. It may be assumed that the oasis did not witness reduction in growth and agriculture production; hence, the number of production wells either remained constant (an unlikely scenario) or increased in number. As regards the latter, temporal groundwater changes may be considered to occur under an increase in groundwater production in Cele, and groundwater level rise may be attributed to increased surface water availability which was in excess during 2010–2017 (Fig. 3).

### Cele Oasis water balance

**Fig. 12:**
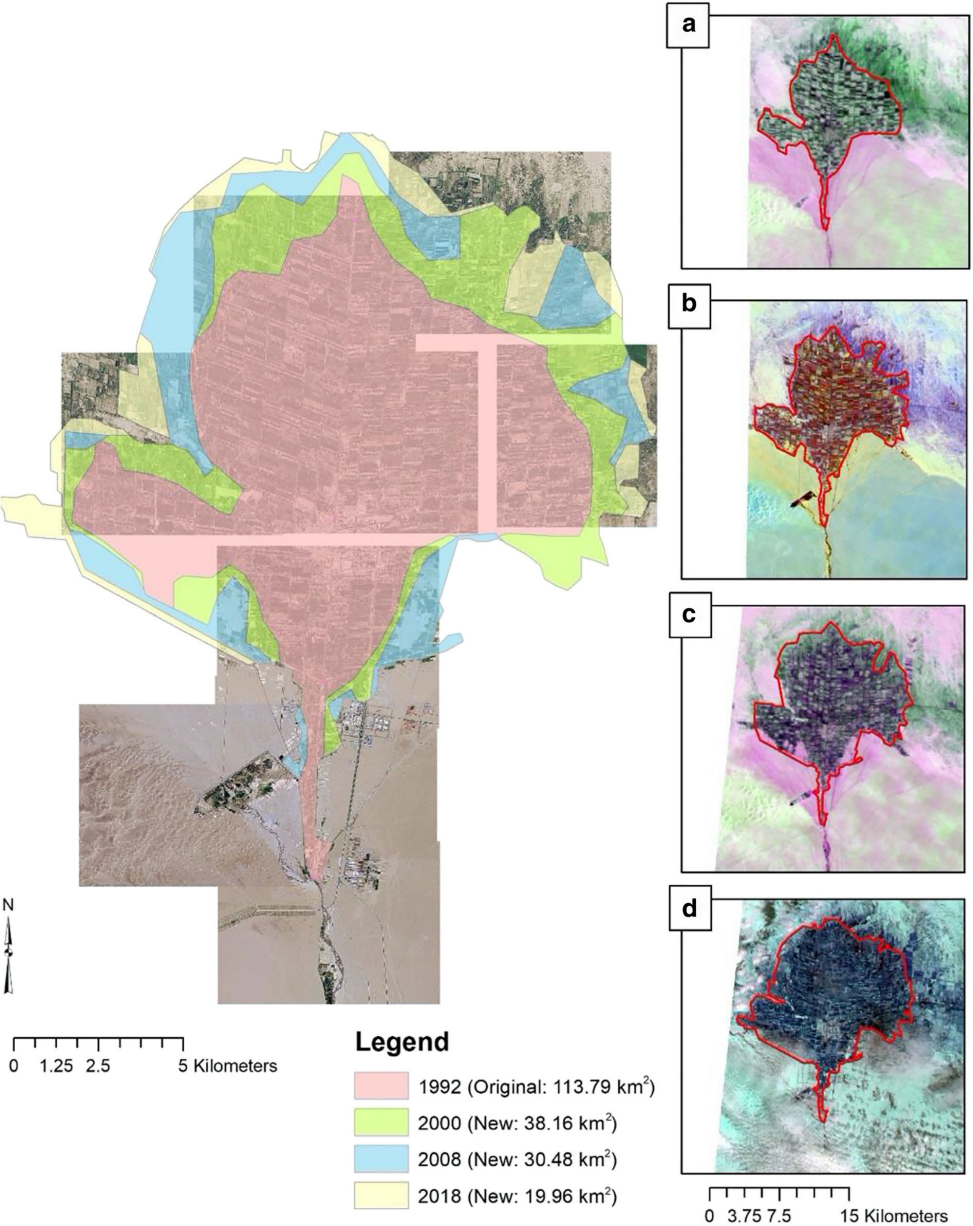
Growth of the Cele Oasis between 1992 and 2018 (Landsat-5, Landsat-7, and Landsat-8 images, courtesy of the US Geological Survey)

The Cele Oasis is an agricultural community with over 90% of its footprint devoted to farming with the remainder a mix of largely residential areas and some local businesses. Historically, the Cele River served as the sole source to farmers for irrigation water; however, in the past few decades, groundwater has been used to supplement surface water supplies and serves as the only source of irrigation water in some areas of Cele. The primary crops in Cele are orchards consisting of fruit-bearing trees and shrubs like walnuts, red dates, and pomegranates. Other crops like wheat and corn are commonly grown within the same space as the orchards creating a mixed crop environment. Many of the fields in Cele are excavated depressions enclosed by earthen berms where irrigation water floods the fields. A secondary form of irrigation is drip which is seeing more use, partly under the guise of water conservation. However, allotment of surface water and withdrawal of groundwater has continued unrestrained by the availability and sustainability of either water source. Over the last quarter of the century, Cele has experienced a 178% growth in size (Fig. 12). Bruelheide et al. (2003), using historic aerial photography from 1956 and satellite imagery from 1999 and 2000, determined that agriculture lands expanded only 14 km^2^. In this investigation, satellite imagery suggests an increase of 38.16 km^2^ between 1992 and 2000. This discrepancy of approximately 24 km^2^ may be attributed to an overestimation of the 1956 bounds owing to (1) the 1956 imagery possibly overestimating a larger agricultural footprint that included interspersed land use mix of natural desert vegetation; (2) a lack of an easily recognizable defined crop plot structure; (3) absence of a central community core area (i.e., present day downtown Cele) that resulted in smaller village cores scattered across the oasis; and (4) a broad smattering of agricultural plots that followed a splay of bifurcated Cele River channels (the Cele canal network did not exist until 1987) mimicking a deltaic depositional pattern. Additionally, the eastern edge of Cele was not included in Bruelheide et al.’s (2003) satellite imagery analysis, yet there is substantial expansion eastward between 1992 and 2000 (Fig. 12). There is a growth rate of 1.77 between 1992 and 2000 which more closely matches the average growth rate of 1.82 of Tarim Basin oases between 1949 and 1990 (Hong et al. 2003). To determine whether these water sources are meeting current irrigation demands and if desired continued growth will overstrain them, a crop water balance analysis was conducted using the growing period March–August.

The first stage of the crop water balance was to determine whether Cele’s largest and oldest source of irrigation water, surface water, was sufficient to meet the irrigation demand. As groundwater serves as a secondary source of water, it was to be determined if groundwater was needed to supplement a surface water deficit or if it could be reserved for future growth and temporary use in areas not presently serviced by Cele’s canal network.

Agricultural plots have been mapped for 2018 that identify plot location, size, and crop type. Additionally, water application rates per the Chinese acre (~ 666 m^2^) are provided for each crop. Mixed crop water application rates are undifferentiated from the parent crop. An analysis of crop water consumption and an overall source water balance was performed. Using ArcGIS (ArcGIS® software by Esri), a water demand spatial distribution was performed.

A mapping of crop type in Cele provides measures of plot surface areas. Farmers’ estimates of water rates per a standard plot size (666 m^2^) were obtained for crop types, but rates for mixed crop plots were not differentiated from plots growing only the primary crop. Table 1 lists the primary crops, mixed crops, and estimated quantity of irrigation water during the growing season. Red dates are the largest crop type in Cele accounting for nearly 60% of all crops and 68% of overall water demand. Walnuts are the second largest crop with 23% crop area and 17.7% water demand. Pomegranate is the third largest crop with 5.8% crop area and 5.8% water demand. These three crops represent approximately 89% of the crop area in Cele and demand nearly 92% of available water resources. The “other” crops category consists of apple, apricot, corn, peach, rose willow, wheat, and others, collectively making up 11% of the crop area and 8.4% of water demand.

**Table 1:** Water volume by crop type in Cele during the primary growing season, March–August

Primary crop	Mixed crop	Water usage (Mar–Aug)	Water rate (m^3^) per 666 m^2^ [a]	Plot area (m^2^)	Percent of total farmed area (%)	Percent of total water demand (%)
Pomegranate	---	3,152,862	400	5,249,514	5.2%	5.3%
	Wheat	406,905		677,496		
Pomegranate	---	35,058,476	513	45,514,512	53.8%	63.0%
	Corn	162,104		210,451		
	Wheat	6,794,496		8,820,925		
Walnut	---	6,365,926	346	12,253,488	20.8%	16.4%
	Corn	219,359		422,235		
	Wheat	4,363,695		8,399,482		
Other		5,240,869	340	10,265,288	11.0%	8.4%

aColumn is measured as meters (m)

**Fig. 13:**
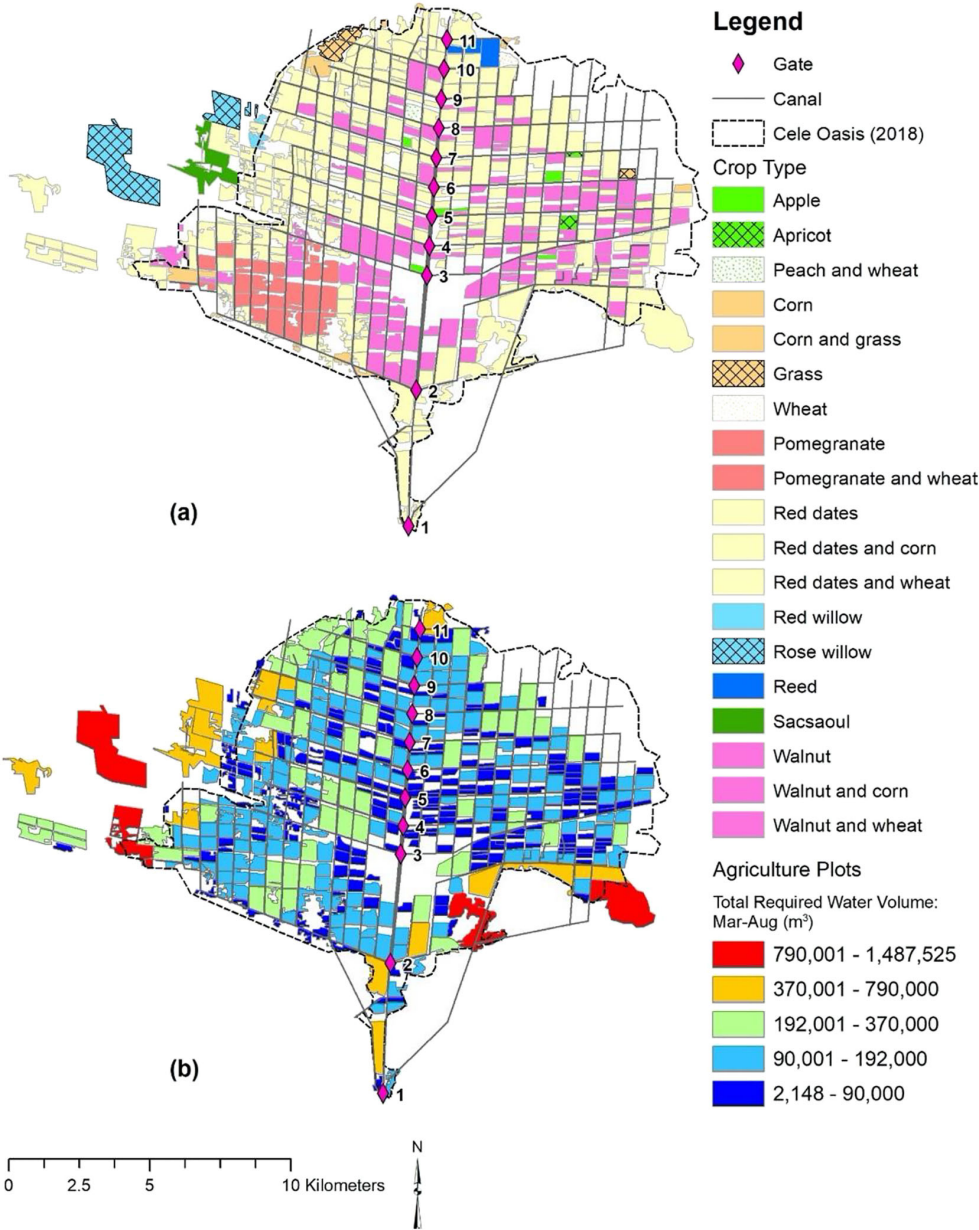
Distribution of crops (a) by type and (b) water demand (Mar–Aug) for 2018

Distribution of crop type is illustrated in Fig. 13(a), as mapped in 2018. Red dates are well distributed throughout Cele. Walnut crops are also reasonably distributed throughout the area, but pomegranate is only grown in Cele’s western region bracketed between two large canal laterals stemming from gates 2 and 3. The remainder of the crop types is scattered about Cele. The large open space south of gate 3 is Cele’s downtown. Empty spaces between the plots outside of canal routes, corresponding to road corridors, are mostly residential areas, open fields, and outskirt businesses. In Cele’s northeast section, farming is not yet substantiated even though the canal network extends into that area. Figure 13(b) depicts the distribution of water demand during March–August based on measures in Table 1.

The total crop water demand during March–August is 6.18 × 10^6^ m^3^. Significant water demand is observed on Cele’s outer boundaries to the southeast, a single area to its north, and along its western border. This disparity with lower water demand among plots within Cele is likely due to the retention of smaller, family-owned plots that have been handed down from generation to generation.

As discussed in the surface water section and illustrated in Fig. 3, surface water can meet the volumetric demands of the agricultural community except during drought. In fact, during the decadal period of 2008–2017 with the exclusion of 2009 when a 5-year drought hit its peak, surface water excess—measured as water diverted from the Cele River into the Cele Oasis canals minus that demanded by agriculture—varied from 0.279 × 10^6^ m^3^ (2014) to 59.8 × 10^6^ m^3^ (2016). As water demanded by agriculture grows, there will be less excess water in future years. Considering 2009 with water inflow figures into the canal system of 49.9 × 10^6^ m^3^, when compared with 2018 crop numbers, there is a perceived deficit of 12.3 × 10^6^ m^3^.

Between 2008 and 2018, Cele expanded by 19.96 km^2^ (see Fig. 12). An analysis of plots added between these years reveals an increased water demand of 9.87 × 10^6^ m^3^, consequently reducing the perceived deficit to 2.43 × 10^6^ m^3^. Does surface water excess directly equate to the expansion of Cele’s agricultural production? When considering this question, agriculture crop demand is known. Added to this is surface water used for drinking water. Measured at gate 1 in 2018, 8.34 × 10^6^ m^3^ was treated for drinking water. This is a significant amount, but in comparison with all the available excesses, surface water would support additional growth. Within Cele’s current boundary, the density of existing plots does not provide additional space between them for any further expansion. Cele must look to its outskirts for expansion. In northeast Cele, canals do exist for such expansion. However, instead of expansion to the northeast, there is an observed expansion to the west where the canal network does not reach.

**Fig. 14:**
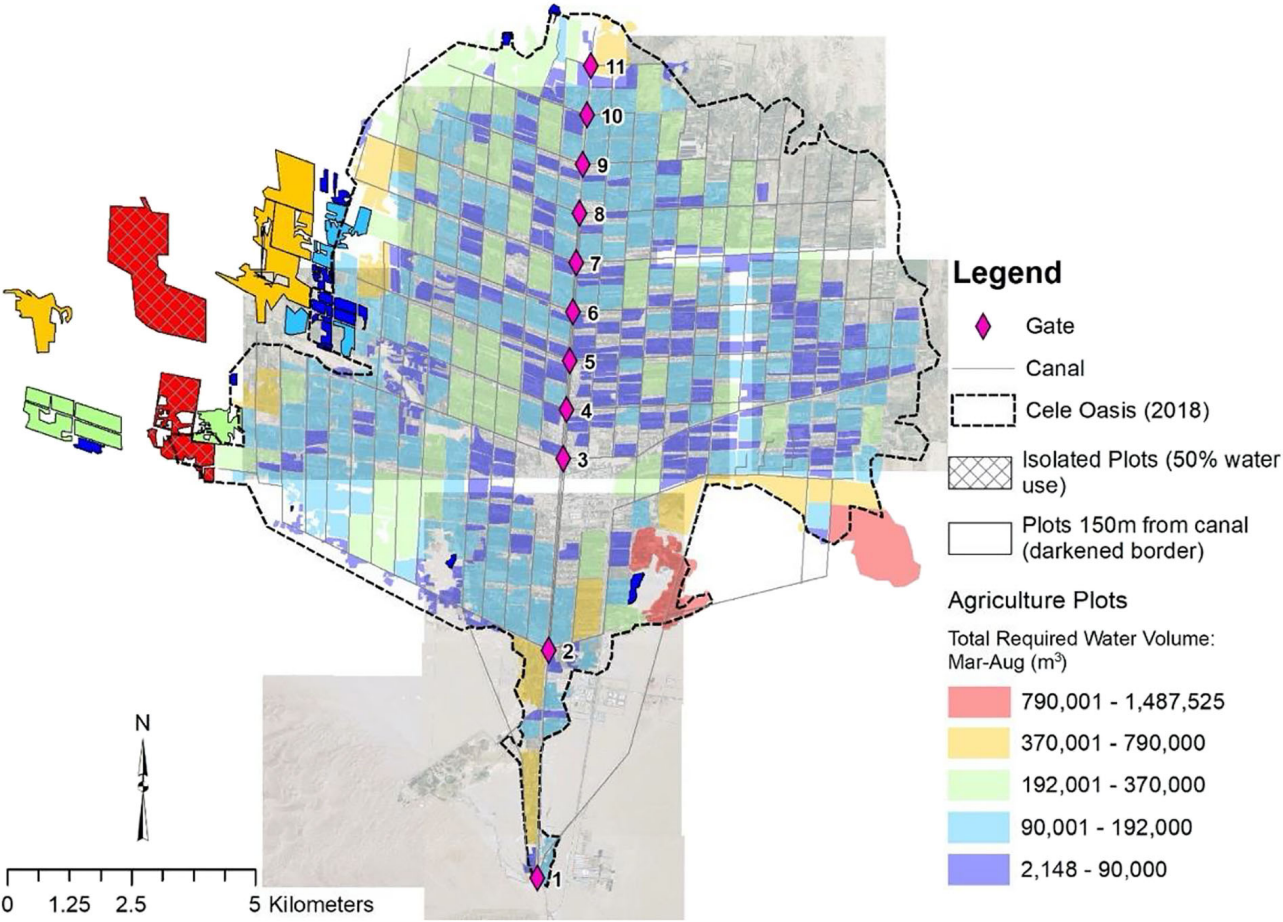
Agricultural plots indicating total water demand during the months of March–August

A conservative measure of > 150 m ($\overline{dist}$_plot to canal_ = 68 m) was used to select plots that are beyond connectivity to the canal network. A total of 36 plots (7% of total plots) met this criterion with nearly 90% clustered on the western border of Cele; however, these isolated plots constitute 12.4% of the land farmed in Cele and 11.7% consumptive amount of surface water demand from the canal network (7.20 × 10^6^ m^3^). These plots have a strong reliance on groundwater. Yet as shown in Fig. 14, the two plots with the highest water demand, representing almost 50% of water use among the isolated plots, are within 1–1.5 km of two major laterals drawing from gates 2 and 3. The average distance of all isolated plots from a canal channel is 550 m; hence, possible extension of laterals from these larger gates may supplement groundwater demand with surface water and conserve underground resources.

## Discussion

Located in the Taklimakan Desert, the Cele Oasis, like many other oases in the region, provides an important role in agricultural production. Situated on the southern outskirts of the desert, Cele receives annual runoff from snowmelt in the Kunlun Mountains. As Cele’s primary water source, surface water has provided irrigation water to farmers, thus allowing Cele to expand its agriculture footprint by 88.6 km^2^ since 1992. Yet this expansion is also in part owing to the use of groundwater for irrigation. Without regard to the finite supply of these resources, Cele has grown unfettered. Yet, there is now a concern to what growth can occur based on these resources.

This analysis reveals that there are excesses of surface water both during and outside the growing season (March–August). Realizing there are additional stressors to this overall excess, such as drinking water demand and losses to evaporation and infiltration, there is still an average of surface water that can support growth of Cele’s agricultural community. A more detailed study is warranted, but an additional 33.51 km^2^ of red dates and 20.82 km^2^ of walnut can be planted based on the following five assumptions: (1) evapotranspiration and infiltration loss (~ 50%) (e.g., reservoir storage, traversing of the Cele River to Cele); (2) a conservative population growth estimate of 10% on the drinking water demand; (3) average excess between the Cele River discharge (gaging station 8 km upstream) and 2018 crop water demand between 2008 and 2015; (4) a similar allocation of environmental flow requirements against total river discharge for river baseflow (1.25%) for ecological/riparian support, 1.09% for sediment transport, and 2.66% of river evaporation based on Xue et al. (2015); and (5) using the land allocation percentages for the two major crops: red dates and walnut (pomegranates are grown only between gate’s 2 and 3 laterals). These estimates assume that use of groundwater for irrigation is restrained to seasons of drought (i.e., utilized in special circumstances) and to support desert ecology, that excess runoff can be retained such as through reservoir storage to supplement low runoff early in the growing period (March–April), and that the Cele canal system works as designed to meet current agriculture demands.

The northeast section of Cele, which has canals but not farmed, is approximately 9.45 km^2^. This would seem to be the first area suggested for agricultural growth; however, soil analyses in this area indicate higher salinity which has resulted in it not being farmed to date. Beyond this remaining canaled area, growth will require modification of the canal system. As the canal infrastructure conveys less volume traversing from gate 2 to 11, the recommendation for growth is in the south of Cele. An engineering analysis is required to determine whether the capacity of the existing laterals from the earlier gates (e.g., gates 2 and 3, thus expanding east and west) or even the main canal can accommodate flows to meet increasing demand. These analyses should include adding storage to the Cele reservoir and possible outflow to new areas of growth targeted in that region. Lastly, measures of periodic groundwater levels on a monthly basis (minimum), calculation of evaporation and infiltration losses from surface water bodies, and continued measure of flow diversions at gate 1 should be routinely conducted.

## Conclusions

Located in an arid environment within the Taklimakan Desert, the Cele oasis supports an agricultural community with farmland production contributing to 97% of its economy. Cele’s agriculture relies on runoff from snowmelt off the Kunlun Mountains into the Cele River and from groundwater beneath the oasis. An unprecedented dataset of Cele River discharge at the entry gate to Cele’s concrete canal network and its reservoir in conjunction with groundwater levels and estimations of water usage by crop type supported an analysis of water availability to Cele. According to these recently available data, Cele receives excess surface water during the high runoff period which is released into the desert. Capture of this excess water, while accounting for necessary contributions to the desert to maintain its ecosystem, would allow Cele’s agricultural community to expand. Choosing Cele’s two most prevalent crop types, Cele could likely add 33.51 km^2^ of red dates and 20.82 km^2^ of walnut. Suggestions are provided as to where these crops would benefit from the existing canal infrastructure. This study highlights the importance of having detailed measurements of an oasis’ critical water resources when attempting to determine potential expansion that, without this information, could possibly overextend these resources to the detriment of the oasis community. Though using the methodologies employed here to develop a procedural roadmap is not part of the study, certainly other oases in the region which face similar challenges may draw from this research to guide their own investigations.
